# A Review of the Role of an Anthocyanin, Cyanidin-3-*O*-*β*-glucoside in Obesity-Related Complications

**DOI:** 10.3390/plants12223889

**Published:** 2023-11-17

**Authors:** Ponnuvel Deepa, Minji Hong, Kandhasamy Sowndhararajan, Songmun Kim

**Affiliations:** 1 School of Natural Resources and Environmental Science, Kangwon National University, Chuncheon 24341, Republic of Korea; taanishadeepa@gmail.com (P.D.); alswl0356@kangwon.ac.kr (M.H.); 2 Department of Botany, Kongunadu Arts and Science College, Coimbatore 641029, India; sowndhar1982@gmail.com

**Keywords:** anthocyanin, cyanidin-3-*O*-*β*-glucoside, cyanidin-3-glucoside, obesity, adipogenesis, adipocyte

## Abstract

Obesity has become a major health issue worldwide and obese individuals possess higher levels of adipose tissue when compared with healthy individuals. Obesity is highly associated with the development of different chronic diseases, such as diabetes, cardiovascular diseases, hypertension, cancers, etc. Previous studies established that anthocyanin compounds play an important role in attenuating obesity-related consequences. Among various anthocyanin compounds, cyanidin-3-*O*-*β*-glucoside (C3G) is the most important component and is widely distributed in various colored edible plant materials, especially berries, cherries, black rice, purple corn, etc. In recent decades, several studies have reported the therapeutical properties of C3G. C3G has various biological properties and health benefits, such as antioxidant, antimicrobial, anti-inflammatory, antidiabetic, anti-obesity, neuroprotective, anticancer, etc. In this review, we summarized the in vitro and in vivo studies in relation to the role of C3G in obesity-related complications. Several mechanistic studies demonstrated that C3G maintains the metabolism of glucose, fatty acids, and lipids by regulating different genes and signaling pathways. It could be concluded that the consumption of C3G protects healthy individuals from obesity-related issues by maintaining body weight and regulating their metabolism and energy balance. This review provides some important signaling pathways/targets of C3G to facilitate the prevention and treatment of obesity, leading to the development of important food supplements.

## 1. Introduction

Obesity is one of the most important health problems worldwide with considerable illness and mortality. According to the WHO, more than 2.8 million deaths occur every year due to obesity-related consequences. In 2016, approx. 1.9 billion adults were obese [1,2]. If secular trends continue, it is estimated that 38% of the world’s adult population will be overweight by 2030 and an additional 20% will be obese exclusively in underdeveloped countries [3]. Genetic mechanisms, endocrine, appetite disorders, and diabetes-associated diseases are major factors that trigger obesity [4,5]. Obesity is a high-risk factor for developing numerous chronic diseases, including diabetes, heart disease, liver disease, and hypertension [6]. Lipid and energetic metabolisms comprise a sequence of biosynthetic and oxidative reactions required for the energy supply and differentiation of adipocytes. In these, lipogenesis activates mitochondrial biogenesis, the production of reactive oxygen species, and inflammatory responses that are highly correlated with obesity [5]. The major causative factor for obesity is the consumption of more calories accompanied by the accumulation of excessive fat. Although lifestyle changes and improving physical activity are the best strategies for preventing obesity, it can be difficult to continue these activities in the long term [7]. Some treatment strategies that can be followed to prevent obesity are thermogenesis of brown adipose tissue, genetics and epigenetics, physical exercise, and diet, including foods with bioactive components [8]. Therefore, developing a novel effective approach to prevent obesity-related complications is essential for the scientific community [6,7].

Anthocyanins are widely found in colored fruits and vegetables. [4]. They are important flavonoid compounds in terms of food as well as medicine due to the availability of numerous structurally different bioactive components [9,10]. Anthocyanin components offer potential health-promoting functions due to their strong antioxidant effects [9,11]. Anthocyanin components are rich in colored fruits, vegetables, and cereals, including berries, cherries, grapes, black beans, purple cabbage, purple corn, black rice, etc. In addition, anthocyanins are generally treated as colorants in beverages, fruit fillings, and dairy products. Hence, anthocyanins play a significant role in the diet of humans [5,12]. Diets supplemented with anthocyanin-rich berries can significantly affect gastrointestinal bacterial communities with respect to obligate anaerobes by decreasing the gastrointestinal luminal oxygen and oxidative stress [13]. In particular, cyanidin and its derivatives can exhibit a protective effect on DNA cleavage [14]. The administration and consumption of cyanidin glycosides are effective in reducing reactive oxygen species (ROS)-mediated cell and tissue damage [15,16].

**Figure 1:**
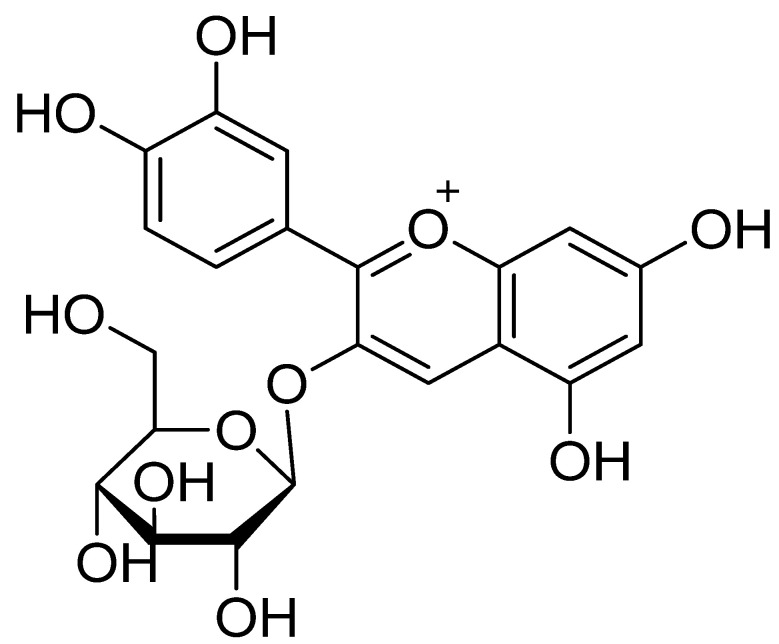
The chemical structure of cyanidin-3-*O*-*β*-glucoside.

In recent times, there has been significant attention in the research of anthocyanin derivatives. Approx. 635 different anthocyanin components have been identified in various plants. In particular, pelargonidin, cyanidin, delphinidin, peonidin, petunidin, and malvidin are important aglycones in foods [5]. Among them, cyanidin-3-*O*-*β*-glucoside (C3G) is an important biologically active component (Figure 1). Numerous studies have confirmed that anthocyanins provide various therapeutic effects, especially C3G, which significantly increases energy expenditure as well as decreases weight gain [10,17]. Further, C3G is considered to be the main component in terms of the bioavailability and active form of cyanidin in human tissues [18]. C3G markedly reduces lipogenesis, oxidative stress, and inflammation, thereby improving the obesity-mediated dysregulation of metabolism. C3G has the potential to speed up lipolysis and thermogenesis and to reduce body fat accumulation. A study found that a master regulator of energy metabolism, namely peroxisome proliferator-activated receptors (PPARs), is activated by C3G [19].

C3G and its metabolites have better absorption and bioavailability, and their interaction with gut microbiota may improve their positive health effects. Several studies on C3G have been published in the past decades. However, no detailed review has been published regarding the role of C3G in obesity-related complications. In this context, the present review aims to discuss the possible therapeutical properties of C3G for preventing and treating obesity based on in vitro and in vivo studies. For this purpose, a literature search was performed in electronic databases using “obesity” as the major term, together with “C3G”, and mainly comprised articles in English. This review mainly focused on the role of C3G in obesity with its underlying mechanisms, leading to the development of potent food supplements for preventing obesity-related health issues.

## 2. The Occurrence and Color Characteristics of C3G

Anthocyanin content in colored fruits and vegetables varies remarkably, according to different species. In addition, numerous factors influence the level of anthocyanin content, such as the cultivar, geographical location, environmental conditions, cultural practices, harvest time and methods, processing and storage conditions, etc. [20,21,22]. Berries are a rich source of anthocyanins, including strawberries, blueberries, blackberries, black elderberries, bilberries, blackcurrants, chokeberries, redcurrants, and raspberries [22]. In these, the highest anthocyanin content is found in elderberries (1.4 g) and chokeberries (1.8 g) per 100 g of product [23,24]. Moreover, purple corn, cherries, plums, pomegranates, eggplant, wine, grapes, and colored vegetables such as black carrots, red cabbage, and purple cauliflower contain appreciable amounts of anthocyanin compounds [24,25]. Among the different anthocyanins, C3G is widely found in colorful berries, blue and red fruits, and vegetables. In particular, pomegranates, blackberries, bilberries, elderberries, and mulberries possess a higher bioavailability of C3G. In addition, C3G is commonly found in black rice, black beans, and purple potatoes [26,27].

The color of the anthocyanin pigments usually ranges from pale yellow to blue, but are primarily responsible for the red, purple, and blue colors of different plant organs. In general, C3G is normally found in vegetables and fruits as a red pigment. However, the pH, light, temperature, and structure play a critical role in the color and stability of anthocyanins. At an alkaline pH, anthocyanins are blue pigments, whereas they are red at an acidic pH. However, these pigments are highly unstable and oxidized into dark brown compounds at alkaline conditions due to degradation [28]. In their structure, the B-ring and the presence of hydroxyl or methoxyl groups also influence the stability of anthocyanins. Further, the presence of an oxonium ion adjacent to carbon 2, metal ions, temperature, light, and oxygen may also influence the stability of the anthocyanin pigments [22,28]. In the flowers and fruits of some plants, the colors may depend on the combined effect of the light absorbance of chlorophylls and anthocyanins [29,30].

## 3. The Chemistry of C3G

Anthocyanins are anthocyanidin glycosides (aglycones). They are formed by a flavylium cation backbone that is hydroxylated in different positions to produce diverse anthocyanidins [29]. Cyanidin is one of the extensively distributed pigments in plants. In these, C3G is the most characterized anthocyanin in edible plants, followed by delphinidin, pelargonidin, and peonidin glucosides. These aglycones differ according to their B-ring substitution pattern [28]. C3G is a monomeric anthocyanin and a water-soluble pigment with a molecular weight of 449.4 g/mol. It is derived from the aglycone cyanidin and is more hydrophilic than cyanidin. C3G consists of an *O*-glycosylated anthocyanidin with two hydroxyls on the third aromatic ring. In acidic conditions, C3G occurs in the flavylium form. On the other hand, in alkaline conditions, C3G occurs in the carbinol form through the hydration of the flavylium cation or in the quinoidal form through the loss of a proton [31]. In plants, C3G is biosynthesized through the flavan-3-ol pathway. Previously, pharmacokinetics studies in humans demonstrated that >20 kinds of C3G metabolites have been identified [32]. The main bioactive metabolites of C3G are protocatechuic acid, phloroglucinaldehyde, vanillic acid, and ferulic acid. Furthermore, the stability of C3G is improved when it is acylated with lauric acid, owing to its ester group [33,34].

## 4. In Vitro Studies on the Role of C3G in Obesity-Related Complications

The excessive consumption of energy-rich foods and an inactive lifestyle are believed to be the main causes of obesity-related insulin resistance and abnormal glucose metabolism. Scientific studies confirmed that anthocyanins may lower the metabolic risks linked with obesity due to their potent antioxidant and anti-inflammatory properties. The dysfunction of adipocytes is highly correlated with the onset of obesity and insulin resistance. It is believed that obesity and the improvement of insulin sensitivity can be prevented by regulating the secretion of adipocytokine and the expression of adipocyte-specific genes. Table 1 shows the mechanisms of C3G for preventing obesity-related complications under different in vitro conditions.

An excessive amount of circulating free fatty acids has been proven to be potentially associated with obesity, insulin resistance, and diabetes. In this context, lipolysis inhibition is the main target for reducing free fatty acids and improving insulin sensitivity. A study reported that C3G effectively suppressed the release of free fatty acids and glycerol from 3T3-L1 adipocytes during hyperglycemia. C3G treatment downregulated the hexosamine biosynthetic pathway by increasing the AMP-activated protein kinase activity, decreasing the glutamine:fructose 6-phosphate aminotransferase activity (GFAT), and reducing the production of cellular UDP-N-acetylglucosamine. Furthermore, C3G downregulated the adipose triglyceride lipase expression by attenuating the forkhead box O1 (FoxO1) transcription factor [45]. C3G efficiently improved insulin resistance in 3T3-L1 adipocytes by upregulating the expression of the glucose transporter type 4 (GLUT4) gene [40].

C3G markedly inhibited basal adipocyte lipolysis in differentiated human fat cells. A mixture of docosahexaenoic acid and C3G reduced the tumor necrosis factor-α (TNF-α) secretion and enhanced insulin-induced lipogenesis [50]. Matsukawa et al. [49] demonstrated that C3G treatment differentiated the 3T3-Ll cells into smaller adipocytes by upregulating the gene expressions of PPARγ and C/EBPα, increasing the adiponectin secretion, decreasing the TNF-α secretion, activating insulin signaling, and increasing the glucose uptake. Further, C3G effectively upregulated the expression of PGC-1α, sirtuin-1 (SIRT1), and the uncoupling protein (UCP)-3 genes in C2C12 myotubes. Jia et al. [19] found that C3G effectively enhanced the uptake of glucose in HepG2 cells as well as C2C12 myotubes. Further, C3G stimulated the hepatic fatty acid oxidation rate. The results revealed that C3G upregulated the main regulators of energy metabolism, i.e., PPARs. In rat adipocytes, C3G increased the secretion of adipocytokines, such as adiponectin and leptin. Further, the adipocyte-specific gene expression was upregulated by C3G treatment without PPARγ activation [35]. In human adipocytes, C3G or cyanidin treatment significantly upregulated the gene expression of adiponectin as well as downregulated plasminogen activator inhibitor-1 and IL-6. C3G or cyanidin treatment also activated the lipid metabolism-associated genes, such as uncoupling protein2, acyl-CoA oxidase1, and perilipin [36].

One of the promising approaches for treating obesity is to convert white adipocytes into brown-like adipocytes. In 3T3-L1 adipocytes, C3G stimulated phenotypic modifications into white adipocytes, such as higher levels of multilocular lipid droplets and mitochondrial content. In addition, C3G treatment increased the expression of the mitochondrial genes in 3T3-L1. Moreover, C3G improved the differentiation of preadipocytes by activating the CCAAT/enhancer-binding protein β (C/EBPβ) via increasing the level of intracellular cyclic adenosine 3′5′-monophosphate (cAMP) [53]. Recently, Han et al. [62] found that Prdm16 bound to the promoter region (−500 to −150 bp) of UCP1 to stimulate its transcription with the presence of C3G in C3H10T12 brown adipose cells, thereby facilitating brown adipose tissue programming. In differentiated palmitate-induced C3H10T1/2 clone8 cells, C3G suppressed the release of adipokines, such as extracellular nicotinamide phosphoribosyltransferase and fibroblast growth factor 21 [54].

A study indicated that C3G treatment significantly regulated protein kinase B (Akt) phosphorylation, the sensitivity of adipocytes to palmitic acid, GLUT-1 and GLUT-4 glucose transporters, and hexokinase-II in palmitic acid-induced human SGBS adipocytes. In addition, the cells pretreated with C3G exhibited a significant reduction in the mRNA levels of pro-inflammatory cytokines (TNF-α, interleukin (IL)-6, IL-8, and monocyte chemoattractant protein-1 (MCP-1)) in palmitic acid-induced human SGBS adipocytes [63]. C3G markedly reduced the accumulation of lipids, PPARγ, and the nuclear factor kappa B (NF-κB) pathways in palmitic acid-induced 3T3-L1 adipocytes, and enhanced insulin sensitivity via the restoration of the insulin receptor substrate 1 (IRS-1)/phosphatidylinositol 3-kinase (PI3K)/Akt pathway. Moreover, C3G improved the mRNA levels of adiponectin in palmitic acid-induced 3T3-L1 and SGBS human adipocytes [60]. C3G treatment efficiently triggered the expression and secretion of adiponectin in 3T3 adipocytes through the transcription factor forkhead box O1 (FoxO1). The authors found that C3G upregulated FoxO1 by enhancing its deacetylation through silent mating type information regulation 2 homolog 1 [47].

In 3T3-L1 adipocytes, a study found that C3G treatment effectively reverted the insulin resistance induced by H_2_O_2_- or TNF-α via the downregulation of the c-Jun N-terminal kinases (JNK) signaling pathway [38]. C3G and its metabolite protocatechuic acid improved the uptake of adipocyte glucose and GLUT4 membrane translocation in human omental adipocytes and 3T3-L1 cells. Further, these components markedly increased the nuclear PPARγ activity and the expression of GLUT4 [42]. In macrophage-conditioned media-treated adipocytes, C3G and pelargonidin-3-O-glucoside suppressed the activation of the NF-κB and JNK pathways by regulating the phosphorylation of IκBα and JNK, respectively [2]. In 3T3-L1 adipocytes, anthocyanins extracted from colored corn blocked the adipocyte differentiation and the accumulation of lipids, and attenuated the transcription of PPAR-γ. In 3T3-L1 cells, black soybean anthocyanins decreased the accumulation of lipids and inhibited the expression of PPARγ [46]. Further, anthocyanins improved inflammation induced by TNF-α and insulin resistance in adipocytes by activating insulin signaling and enhanced translocation of GLUT4 [52].

Further, AMPK plays a key role in preventing hepatic lipid metabolism by regulating the downstream acetyl CoA carboxylase and carnitine palmitoyl transferase 1 pathways. A study indicated that C3G regulated hepatic lipid homeostasis through an AMPK-dependent signaling pathway [44]. In 3T3-L1 preadipocytes, cyanidin treatment suppressed adipogenesis via the downregulation of gene expressions, such as PPARγ, C/EBPα, adiponectin, and aP2. In addition, intracellular Ca^2+^ was increased in the cyanidin-stimulated cells [66]. Cyanidin-3-rutinoside (C3R) significantly upregulated the uptake of glucose and the expression of plasma membrane glucose transporter type 4 (GLUT4) by triggering the PI3K/Akt pathways [51]. In mesenteric adipose tissue-cultured medium-induced RAW 264.7 cells, cyanidin and C3G markedly inhibited the migration and production of inflammatory chemokines, such as MCP-1 and MRP-2 [37]. Zhang et al. [59] investigated the effect of anthocyanin-rich water extracts obtained from 20 different purple corn genotypes on RAW 264.7 macrophages and 3T3-L1 adipocytes. These extracts efficiently decreased the production of the pro-inflammatory mediators, regulated the diabetes-associated key enzymes, and improved insulin sensitivity. In the early phase of adipogenesis, C3G exposure markedly suppressed the expression of (C/EBPβ), PPARɣ, and FAS, and activated the AMPK pathway when compared to late-phase exposure [68].

Saulite et al. [58] investigated the effect of malvidin, cyanidin, and delphinidin against adipose tissue-derived human mesenchymal stem cells. The results revealed that delphinidin suppressed adipogenesis and decreased fatty acid-binding protein 4 (FABP4) and the adiponectin genes. Malvidin stimulated a higher level of calcium accumulation in the cells. Further, the osteocyte-specific gene BMP-2 and Runx-2 expression were upregulated in addition to the stimulation of BMP-2 secretion by malvidin. According to suitable stimuli, adipocytes can be proliferated and differentiated. The important transcription factor responsible for lipogenesis is the carbohydrate response element-binding protein (ChREBP) chiefly found in lipogenic organs. Pompei et al. [43] found that cyanidin diminished the differentiation by 20% as well as the ChREBP expression in preadipocytes. The authors suggested that cyanidin exhibited an inhibitory activity on adipogenesis by interfering with the extracellular matrix [43]. Takahashi et al. [64] reported that C3G showed significant induction in adipocyte differentiation in human amniotic epithelial cells.

Pancreatic lipase is one of the most essential digestive enzymes for metabolism as well as for the absorption of triglycerides to monoglycerides and free fatty acids. In the body, the level of total cholesterol is decreased when inhibiting the lipase enzyme activity [69]. In this context, different authors investigated the inhibitory potential of C3G against lipase enzymes. Their studies showed that C3G markedly inhibited the pancreatic lipase activity with IC_50_ values of 188.28 µM [57] and 0.268 mg/mL [67]. A recent study demonstrated that the development of insulin fibrils was attenuated by anthocyanins, such as cyanidin, C3G, C3R, malvidin, and malvidin-3-glucoside [65]. In another study, the results exhibited that C3R effectively inhibited pancreatic lipase (IC_50_ at 59.4 μM). Further, C3R showed a considerable reduction in the cholesterol uptake in free cholesterol as well as mixed micelles in addition to the suppression of the NPC1L1 expression in Caco-2 cells [56]. Chen et al. [70] found that C3G from *Aronia melanocarpa* exhibited the strongest inhibitory activity against α-amylase and lipase. However, cyanidin 3-galactoside, cyanidin-3-arabinoside, and cyanidin 3-xyloside did not show any effect on the α-amylase and lipase enzymes. Further, Xie et al. [71] suggested that the structures of the B-ring and glycosyl group were mainly associated with the inhibitory potentials of monomeric anthocyanins.

## 5. In Vivo Studies on the Role of C3G in Obesity-Related Complications

Similar to the in vitro studies, numerous in vivo studies have been carried out to determine the effect of C3G on obesity-associated mechanisms (Table 2). The C3G-rich purple corn color effectively reduced body weight gain as well as the weights of white and brown adipose tissue in high-fat (HF) diet-induced mice. Further, the purple corn color downregulated the mRNA levels of the enzymes responsible for the synthesis of fatty acid triacylglycerol and reduced the mRNA level of sterol regulatory element-binding protein-1 in white adipose tissue [72]. In another study, the dietary intake of anthocyanin upregulated the adiponectin expression in white adipose tissue and suggested that the activation of AMPK was a possible mechanism for these changes [35].

It is well known that brown adipose tissue burns energy to generate heat. The previous studies found that C3G can improve the thermogenic ability of brown adipose tissue. Han et al. [62] evaluated the effect of C3G on the UCP1 gene expression in db/db mice induced with a HF, high-fructose diet and found that Prdm16 is directly bound to the promoter region of UCP1. In another study, C3G efficiently regulated the transcription of UCP1 in brown adipose tissue as well as in subcutaneous white adipose tissue by enhancing mitochondrial number and function [17]. In the diet-induced obesity mice model, the purple corn cob extract treatment upregulated the M2 markers (ArgI, Fizz1, and TGFβ) and downregulated the inflammatory mediators (TNF-α, IL-6, IL-1β, and COX-2) by suppressing NF-kB signaling [94]. Jia et al. [19] reported that the dietary supplementation of C3G upregulated the PPARs in the HF-diet-induced mice. In the KK-Ay mice, C3G significantly enhanced obesity and triglyceride metabolism by partly activating lipoprotein lipase in the plasma and skeletal muscle. The inhibition of lipoprotein lipase (LPL) in adipose tissue following the activation of pAMPK might have been attributed to the inhibition of lipoprotein lipase [41]. Guo et al. [76] reported that C3G regulated the signaling pathway, c-Jun N-terminal kinase/foxO1, and its associated inflammatory adipocytokines. Ren-Qiang et al. [78] also reported that C3G diminished obesity-related dyslipidemia and insulin resistance in HF-diet-induced rats by enhancing the level of serum adiponectin. Biswas et al. [83] reported that C3G supplementation in the diet reduced weight gain, and improved food intake and metabolism during obesity.

The combination of peptides and C3G markedly improved the glucose uptake with or without insulin. In addition, this combination suppressed the expression of the angiotensin II receptor, type 1 (AGTR-1), and activated the insulin receptor substrate 1 and GLUT4 expressions [84]. In diet-induced obese mice, the mixture of C3G and peptides efficiently decreased systolic as well as diastolic blood pressure. Further, this combination significantly reduced body fat levels and enhanced glucose tolerance [87]. In diet-induced obese mice, the combination of C3G and peptides regulated the expression of the genes associated with glucose metabolism [91]. The administration of anthocyanins from purple corn remarkably reduced body weight gain, fat mass, total cholesterol, and triglyceride levels in the HF-diet-induced mice. The data demonstrated that the anthocyanin extract suppressed the expression of PPARγ, C/EBPα, and SREBP-1c, subsequently activating the expression of PPARα, PGC1α, PRDM16, and FGF21 [4].

Titta et al. [39] demonstrated that the anthocyanin-rich extract from Moro oranges marginally affected the accumulation of fats in C57/Bl6 mice induced with a HF diet. Anthocyanin black carrots fermented with *Aspergillus oryzae* inhibited lipid and glucose metabolism via the activation of hepatic insulin signaling and the AMPK pathways in OVX rats [48]. A study demonstrated that anthocyanins from black rice, black soybeans, and purple corn alleviated oxidative stress and inflammation-related obesity in HF-diet-induced mice by suppressing the expression of TNFα, IL-6, inducible nitric oxide synthase (iNOS), and NF-κB, and increasing the hepatic superoxide dismutase (SOD) and glutathione peroxidase (GPx) activities [82]. Blackberries containing 87% of the C3G diet downregulated the expression levels of hepatic NF-κB and cyclooxygenase-2 in ovariectomized rats [75]. A C3G-enriched *Aronia melanocarpa* extract inhibited adipogenesis by downregulating the expressions of the CCAAT/enhancer-binding protein, PPAR, sterol regulatory element-binding protein-1c, PPARγ coactivator-1, acetyl-CoA carboxylase 1, ATP-citrate lyase, fatty acid synthase, and adipocyte protein 2 [88]. The anthocyanin-rich extract from aronia fruit reduced the accumulation of visceral fat and hyperglycemia via the inhibition of pancreatic lipase activity and the adsorption of intestinal lipids [95]. In HF-diet-induced rats, C3G isolated from *Aronia melanocarpa* treatment expressively decreased the levels of TNF-α, IL-6, and IL-1β in the serum. Further, C3G improved HFD-induced obesity by activating AMPK and suppressed HFD-induced inflammation by activating the phosphorylation of STAT3 and suppressing NF-κB p65 in the nucleus [90].

Anthocyanins, such as C3G, C3R, and pelargonidin-3-glucoside, isolated from Chinese mulberry significantly inhibited body weight gain, reduced insulin resistance, lowered the size of adipocytes, and attenuated the accumulation of lipids and the secretion of leptin [77]. The supplementation of cyanidin and delphinidin effectively controlled overweight, obesity, and type 2 diabetes by modulating the activity of inflammatory mediators, oxidative stress, and NF-κB/JNK activation [85]. Esposito et al. [79] suggested that the gut microbiome and the kind of anthocyanin aglycone moiety could influence the preventive role of anthocyanins in obesity and their associated insulin resistance. The supplementation of cyanidin- and delphinidin-rich extracts also exhibited beneficial effects on unhealthy diets [92]. The administration of C3G from Saskatoon berries diminished liver steatosis, insulin resistance, and chronic inflammation in HF and high-sucrose-induced mice [89].

The improvement in the thermogenesis of brown adipose tissue is an efficient strategy for alleviating obesity. A study exhibited that C3G improved the energy expenditure and thermogenesis of brown adipose tissue in mice induced with HF diet obesity. Additionally, C3G upregulated the UCP1 expression as well as other thermogenic genes in inguinal white adipose tissue and brown adipose tissue [86]. In db/db mice, C3G treatment restored the endothelium-dependent relaxation of the aorta [47]. C3G controlled the adipokine expression in brown adipose tissue in high-fat-cholesterol diet-induced mice [54]. A recent study demonstrated that treatment with casein/C3G nanoparticles ameliorated the HFD-induced accumulation of fats and liver oxidative stress in C57BL/6 mice [93]. In a recent study, Meleleo et al. [96] demonstrated that anthocyanins, such as cyanidin, C3G quercetin, and quercetin-*O*-glucoside, interacted with planar lipid membranes and formed conductive units.

## 6. Conclusions and Future Perspectives

C3G is an important anthocyanin compound and is widely found in various fruits and vegetables. Previous studies established that C3G supplementation offers numerous health benefits to the consumer. In this review, we discussed the effect of C3G treatment on obesity-associated mechanisms under different in vitro and in vivo models. The mechanisms preventing obesity highly depend on their capacity to control the consumption of food and energy metabolism and improve inflammatory response, insulin resistance, and glucose metabolism. In addition, C3G can effectively inhibit the pancreatic lipase enzyme. A variety of pathways and gene expressions play a crucial role in preventing obesity-related consequences. In particular, AMPK is an important pathway, attributed to the prevention of hepatic lipid metabolism, and PPARs play a key role in energy metabolism. The IRS-1/PI3K/Akt pathway and GLUT4 gene expression are mainly responsible for insulin sensitivity and the NF-κB signaling pathway is for inflammatory response. The published papers clearly showed that C3G could be a potential agent for facilitating the prevention and treatment of obesity-associated complications owing to its multi-targeted activities. Although C3G can effectively attenuate obesity-associated complications, further clinical studies leading to the development of C3G-enriched functional foods are required.

## Figures and Tables

**Table 1:** The effects of cyanidin-3-*O*-*β*-glucoside on the obesity-associated mechanisms under in vitro conditions.

Model	Concentration	Mechanism(s)	Year of Publication	References
Rat adipocytes	100 µM	Enhanced adiponectin and leptin secretion Upregulated adipocyte-specific gene expressions	2004	[35]
Human preadipocytes	100 µM	Upregulated adiponectin and downregulated PAI-1 and IL-6	2006	[36]
3T3-L1 adipocytes and RAW 264.7 cells	10, 50, and 100 μM	Inhibited the release of MCP-1 and MRP-2	2007	[37]
H_2_O_2_- and TNF-α-induced insulin resistance in 3T3-L1 adipocytes	10, 20, and 40 µM	Inhibited the JNK signal pathway	2008	[38]
Primary brown preadipocytes	100 µM	Decreased the insulin-induced ROS production	2010	[39]
3T3-L1 adipocytes	20 and 100 μM	Upregulated the GLUT4 gene expression	2011	[40]
Skeletal muscle cells and adipocytes from female KK-Ay mice	10, 50, and 100 μmol/L	Activated LPL in plasma and skeletal muscle Inhibited LPL in adipose tissue by activating pAMPK	2011	[41]
Human omental adipocytes and 3T3-L1 cells.	Human omental adipocytes—50 and 100 µmol/L; 3T3-L1 cells—10 and 100 µmol/L	Increased the adipocyte glucose uptake and GLUT4 translocation Increased nuclear PPARγ, adiponectin, and the GLUT4 expressions	2011	[42]
Preadipocytes from human adipose explant tissue	50 μM	Inhibited the expression of ChREBP	2012	[43]
Human HepG2 cells	100 μM	Increased cellular AMPK activity. Induced AMPK downstream target ACC phosphorylation and inactivation Decreased malonyl CoA contents	2012	[44]
3T3-L1 adipocytes	50 µM	Increased the activity of AMPK and decreased the activity of GFAT Decreased the expression of adipose triglyceride lipase by attenuating the O-glycosylation of the transcription factor FoxO1	2012	[45]
Preadipocyte 3T3-L1 cells	Black soybean anthocyanins; 12.5 and 50 µg/mL	Inhibited the proliferation of preconfluent preadipocytes and maturing postconfluent adipocytes Reduced the number of viable cells Increased epinephrine-induced lipolysis Reduced lipid accumulation and suppressed the expression of PPARγ	2012	[46]
3T3 adipocytes	12.5, 25, and 50 µM	Induced the adiponectin expression and secretion by Foxo1	2014	[47]
3T3-L1 fibroblasts	5 or 20 µM	Decreased fat accumulation in 3T3-L1 adipocytes by increasing CPT-1 Decreased FAS and the SREBP-1c expression	2015	[48]
3T3-L1 adipocytes	20 and 100 μM	Increased the PPARγ and C/EBPα gene expressions Increased adiponectin secretion, decreased TNF-α secretion, activated insulin signaling, and increased the glucose uptake Increased the expression of the PGC-1α, SIRT1, and UCP-3 genes	2015	[49]
Primary human preadipocytes	100 μM	Decreased the TNFα secretion and increased insulin-stimulated lipogenesis	2016	[50]
3T3-L1 adipocytes	10 and 50 μM	Activated the PI3K/Akt pathways	2017	[51]
3T3-L1 adipocytes	50 μM	Prevented adipocyte differentiation, lipid accumulation, and reduced PPAR-γ activity Activated insulin signaling and enhanced GLUT4 translocation	2017	[52]
3T3-L1 adipocytes	50 and 100 μM	Increased cAMP levels and the expression of the mitochondrial genes (TFAM, SOD2, UCP-1 and UCP-2), UCP-1 protein, and beige adipocyte markers (CITED1 and TBX1)	2017	[53]
Brown adipose tissue C3H10T1/2 clone 8 cells induced by palmitate	Isolated from mulberry; 100 and 200 μg/mL	Inhibited the release of adipokines Reduced lipid accumulation	2018	[54]
Palmitic acid-induced 3T3-L1 adipocytes	Anthocyanin-rich extract; 10 and 20 μg/mL	Inhibited the activation of the NF-κB pathway Regulated the PI3K/Akt, GLUT-1, and adiponectin mRNA levels	2019	[55]
Macrophage–adipocyte interaction, using mono- and co-culture	1.0 mg/mL of the anthocyanin-rich extracts of purple and red maize or 50 μM of pure anthocyanins	Reduced the production of pro-inflammatory cytokines Inhibited the activation of NF-κB and JNK	2019	[2]
Pancreatic lipase and cholesterol esterase Caco-2 cells	12.5–100 μM	Inhibited pancreatic lipase and pancreatic cholesterol esterase activity Inhibited the formation of cholesterol micelles Reduced cholesterol uptake Suppressed the mRNA expression of NPC1L1	2019	[56]
Pancreatic lipase activity	50 to 350 µM	Cyanidin—IC50 at 28.29 µM C3G—IC50 at 188.28 µM	2019	[57]
Human adipose tissue	25 μM	Inhibited adipogenesis and downregulated adiponectin genes Induced a higher accumulation of calcium deposits Upregulated the osteocyte-specific gene BMP-2, Runx-2 expression, and BMP-2 secretion	2019	[58]
RAW 264.7 macrophages and 3T3-L1 adipocytes	Anthocyanin-rich water extracts (PMWs) from purple maize; 1 mg/mL	Downregulated the production of pro-inflammatory mediators and improved insulin sensitivity	2019	[59]
3T3-L1 hypertrophic adipocytes exposed to palmitic acid	5–10 μM	Reduced lipid accumulation and the PPARγ and NF-κB pathways Improved the adiponectin mRNA levels Ameliorated adipose tissue dysfunction	2020	[60]
Palmitic acid-induced proximal tubular cells	2, 10, and 20 μM	Inhibited the activation of NF-kB and the expression of inflammatory cytokine	2020	[61]
HepG2 cells and C2C12 myotubes	10 and 50 μM	Increased glucose uptake Induced the rate of hepatic fatty acid oxidation Activated PPARs with the highest affinity for PPARα	2020	[19]
C3H/10T1/2 brown adipose cells	10–40 µM	Regulated the UCP1 protein	2021	[62]
Human SGBS adipocytes and murine 3T3-L1 cells.	1–20 μM	Reduced mRNA levels and prevented lipotoxicity in dysfunctional adipocytes	2021	[63]
Human amniotic epithelial cells (hAECs)	20 µM	In differentially expressed genes, 109 genes were upregulated and 232 were downregulated.	2021	[64]
3T3-L1 preadipocytes	50–200 μM	Reduced the formation of insulin fibrils Attenuated insulin fibril-induced cytotoxicity	2022	[65]
3T3-L1 preadipocytes	30–100 μM	Downregulated PPARγ, C/EBPα, adiponectin, and aP2 Inhibited adipocyte formation	2023	[66]
Pancreatic lipase inhibitory assay	0.05 to 0.35 mg/mL	C3G inhibited pancreatic lipase at IC50—0.268 mg/mL	2023	[67]
3T3-L1 preadipocytes	5 and 10 μM	Reduced the C/EBPβ, PPARγ, FAS expressions, and activated the AMPK pathway in the early phase of adipogenesis	2023	[68]

**Table 2:** The effect of cyanidin-3-*O*-*β*-glucoside on the obesity-associated mechanisms under in vivo conditions.

Model	Dose	Mechanism(s)	Year of Publication	References
HFD-induced C57BL/6J mice	Purple corn extract; 2 g/kg diet	Suppressed the mRNA levels of the enzymes involved in fatty acid and triacylglycerol synthesis Lowered the SREBP-1 level in white adipose tissue	2003	[72]
HFD-induced C57/Bl6 mice	90 mg/kg/day	Reduced body weight gain and fat accumulation	2010	[39]
HFD-induced C57BLK/6J mice	C3G-rich grape pomace extract; 250 mg/kg b.w. per day	Lowered plasma C-reactive protein levels	2010	[73]
KK-Ay mice	10, 50, and 100 μmol/L; 1 g/kg	Reduced obesity, the accumulation of fat, and the plasma triglyceride levels	2011	[41]
HFD-induced C57BL/6 mice	3G-rich black soybean seed coat extract for 14 weeks	Suppressed fat accumulation, reduced the plasma glucose level, and enhanced insulin sensitivity Upregulated UCP-1 in brown adipose tissue and UCP-2 in white adipose tissue Downregulated inflammatory cytokines, TNF-α, and MCP-1 in white adipose tissue	2011	[74]
Sprague-Dawley rats—ovariectomized (OVX)	5% and 10% (*w*/*w*) black berries	Decreased hepatic NF-κB, and the cyclooxygenase-2 expression levels	2012	[75]
HFD-fed C57BL/6J mice and db/db and db/+ mice	0.2%	Reduced the white adipose tissue mRNA levels and inflammatory cytokines Decreased the c-JNK activation and promoted the phosphorylation of FoxO1	2012	[76]
HFD-fed C57BL/6 mice	40 and 200 mg/kg food for 12 weeks	Inhibited body weight gain, reduced insulin resistance, lowered the size of adipocytes, and reduced lipid accumulation and leptin secretion	2013	[77]
HFD-induced rats	100 mg/kg	Decreased body weight, visceral adiposity, the average feed efficiency ratio, triglyceride, total cholesterol, low density lipoprotein cholesterol, fasting glucose, and the insulin resistance index Normalized the serum adiponectin and high-density lipoprotein cholesterol levels	2014	[78]
db/db mice	2 g/kg diet	Restored endothelium-dependent relaxation of the aorta	2014	[47]
Estrogen-deficient animals with diet-induced obesity in OVX rats	Black carrot extract; 2% for 12 weeks	Reduced fat mass and weight gain Normalized HOMA-IR Prevented the increase in the serum total and LDL cholesterol and triglycerides Upregulated the gene expressions of CPT-1 and PPAR-α and downregulated the expressions of FAS and SREBP-1c	2015	[48]
HFD-induced C57BL/6J mice	Cyanidin-based anthocyanin-rich blackcurrant extract; 1% for 8 weeks	Reduced body weight gain and improved glucose metabolism	2015	[79]
HFD-induced C57BL/6J mice	Anthocyanin-rich black elderberry extract; 20–40 mg and 100–200 mg/kg b.w. for 16 weeks	Reduced liver weights, serum triglycerides, and MCP-1 Decreased serum insulin and TNFα Attenuated the mRNA level of FAS,PARγ2, and liver cholesterol	2015	[80]
Diabetes model in KK-Ay mice	C3G-rich aronia juice; free intake	Reduced body weights and blood glucose levels Reduced weights of white adipose tissues	2016	[81]
HFD-induced mice	Purple corn anthocyanin; 200 mg/kg	Increased the fecal butyric acid levels, elevated hepatic SOD and GPx activities, and decreased lipid peroxidation Downregulated the expression levels of TNFα, IL-6, iNOS, and NF-κB	2017	[82]
db/db mice	1 mg/mL for 16 weeks	Increased energy expenditure, limited weight gain, maintained glucose homeostasis, reversed hepatic steatosis, improved cold tolerance, and enhanced BAT activity Regulated the transcription of UCP1	2017	[17]
C57BL/6 J mice fed with a high-fat high-cholesterol diet	200 mg/kg	Regulated the activation of brown adipose tissue and the expression of adipokines Alleviated diet-induced fatty liver	2018	[54]
HFD-induced obese mice	Haskap Berry; 0.192% C3G-rich extract	Decreased body weight gain Decreased glucose excursion and exhibited lower glycemia	2018	[83]
Human primary myotubes derived from obese and obese T2DM participants	10 and 100 μM	Enhanced glucose uptake Downregulated the expression of AGTR-1, and upregulated the expression of IRS-1 and GLUT4	2018	[84]
HFD-fed C57BL/6J mice	AC-rich blend; 2, 20, or 40 mg/kg body weight	Mitigated obesity, dyslipidemia, and insulin resistance Attenuated increased liver lipid deposition and inflammation Suppressed oxidative stress, NF-κB and JNK activation, and PTP1B overexpression	2018	[85]
HF and high-fructose diet-induced mice	1 mg/mL	Enhanced energy expenditure and thermogenic capacity Increased the expression of UCP1 and other thermogenic genes	2018	[86]
Diet-induced mouse model	0.02 g/kg BW/ day	Reduced systolic and diastolic blood pressure Reduced the percentage of body fat and improved glucose tolerance	2019	[87]
HFD-induced C57BL/6N mice	*Aronia melanocarpa* extract (70% ethanol extract); 50, 100, and 200 mg/kg body weight/day	Reduced the serum levels of leptin, insulin, triglyceride, total cholesterol, and LDL cholesterol Decreased the CCAAT/enhancer-binding protein, PPAR, sterol regulatory element-binding protein-1c, PPARγ coactivator-1, acetyl-CoA carboxylase 1, ATP-citrate lyase, fatty acid synthase, and adipocyte protein mRNA expressions	2019	[88]
C57BL/6 J mice	50 mg/day/body weight for 8 weeks	Reduced plasma and hepatic triglycerides, glucose tolerance, and adiposity	2020	[19]
HFD-induced C57BL/6J mice	Lingonberry (5% *w*/*w*) and its anthocyanin; C3G for 12 weeks	Enhanced the plasma lipid and glucose profiles and reduced the plasma inflammatory cytokine levels	2020	[61]
HF and high-fructose diet-induced db/db mice	1 mg/mL	Increased the expression of the UCP1 gene of BAT	2021	[62]
HFD-induced mice	Purple corn anthocyanin; 400 mg/kg	Downregulated the expression of PPARγ, C/EBPα, and SREBP-1c Upregulated the expression of PPARα, PGC1α, PRDM16, and FGF21 Promoted the hepatic AMPK activity	2021	[4]
High-fat high-sucrose diet-indued mice	45.2 mg/kg for 10 weeks	Attenuated liver steatosis, insulin resistance, and chronic inflammation	2021	[89]
HFD Sprague-Dawley rats	Isolated from *Aronia melanocarpa*, Haicheng, China; 100 and 200 mg/kg bw/day) for 8 weeks.	Inhibited body weight gain and reduced serum lipids and fat accumulation Decreased the levels of TNF-α, IL-6, and IL-1β in serum Alleviated obesity by promoting the phosphorylation of AMPK Promoted the phosphorylation of STAT3 and suppressed NF-κB p65	2021	[90]
HF-high-carbohydrate diet-induced obesity model	0.02 g/kg BW/d	Downregulated AGTR-1 and upregulated the GLUT4 mRNA expression	2022	[91]
High-fat meal in healthy humans	Cyanidin- and delphinidin-rich extract (1 g consisted of 150 mg bilberry extract, 230 mg black currant extract, and 620 mg black rice extract)	Mitigated endotoxemia and reduced increases in plasma LPS and the LPS-binding protein Attenuated plasma glucose and triglyceride increases, TNFα and NOX4 upregulation, and JNK1/2 activation	2022	[92]
HFD-fed C57BL/6 mice	Casein/C3G nanoparticles	Ameliorated fat accumulation and liver oxidative stress	2023	[93]

## Data Availability

Not applicable.

## Abbreviations

ACC	Acetyl-CoA carboxylase
AGTR-1	Angiotensin II Receptor Type 1
Akt	Protein Kinase B
AMPK	Adenosine monophosphate-activated protein kinase
aP2	Adipocyte Protein 2
ATP	Adenosine triphosphate
BMP-2	Bone Morphogenetic Protein-2
C/EBPα	CCAAT/enhancer-binding protein α
C3G	Cyanidin-3-*O*-*β*-glucoside
C3R	Cyanidin-3-rutinoside
cAMP	Cyclic adenosine 3′,5′-monophosphate
ChREBP	Carbohydrate response element-binding protein
COX-2	Cyclooxygenase 2
CPT-1	Carnitine palmitoyltransferase I
FABP4	Fatty acid-binding protein 4
FAS	Fatty acid synthase
FGF21	Fibroblast growth factor 21 i
FoxO1	Forkhead box protein O1
GFAT	Glutamine:fructose-6-phosphate aminotransferase
GLUT4	Glucose transporter type 4
GPx	Glutathione peroxidase
H_2_O_2_	Hydrogen peroxide
hAECs	Human aortic endothelial cells
HFD	High-fat diet
IL-1β	Interleukin-1 beta
IL-6	Interleukin-6
iNOS	Inducible nitric oxide synthase
IRS-1	Insulin receptor substrate 1
IκBα	Inhibitor of nuclear factor kappa B
JNK	c-Jun N-terminal kinase
LPL	Lipoprotein lipase
malonyl CoA	Malonyl coenzyme A
MCP-1	Monocyte chemoattractant protein-1
mRNA	Messenger RNA
MRP-2	Macrophage inflammatory protein-related protein-2
NF-κB	Nuclear factor kappa B
NPC1L1	Niemann-Pick C1-Like 1
OVX	Ovariectomy
PAI-1	Plasminogen activator inhibitor-1
PGC1α	Peroxisome proliferator-activated receptor-γ coactivator 1-α
PI3K	Phosphoinositide 3-kinase
PPARs	Peroxisome proliferator-activated receptors
PPARα	Peroxisome proliferator-activated receptor alpha
PPARγ	Peroxisome proliferator-activated receptor gammaa
PRDM16	PR/SET Domain 16
PTP1B	Protein tyrosine phosphatase 1B
ROS	Reactive oxygen species
Runx-2	Runt-related transcription factor 2
SIRT1	Sirtuin1
SOD	Superoxide dismutase
SOD2	Superoxide dismutase-2
SREBP-1c	Sterol regulatory element-binding protein 1c
STAT3	Signal transducer and activator of transcription 3
TFAM	Mitochondrial transcription factor A
TNF-α	Tumor necrosis factor alpha
UCP	Uncoupling protein
WHO	World Health Organization
